# Morphological and Molecular Characterization of *Tylenchorhynchus Clarus* Allen, 1955 and *T. Zeae* Sethi & Swarup, 1968 (Rhabditida: Telotylenchidae) from Iraq

**DOI:** 10.2478/jofnem-2022-0043

**Published:** 2022-10-19

**Authors:** Ahmed Malik Jumaah, Sedighe Azimi

**Affiliations:** 1 Department of Plant Protection, Faculty of Agriculture, Shahid Chamran University of Ahvaz, Ahvaz, Iran

**Keywords:** D2–D3 LSU, ITS rDNA, Misan province, morphology, morphometric data, phylogeny, pumpkin, sugarcane, Tylenchorhynchus

## Abstract

During a survey on the biodiversity of plant-parasitic nematodes in Misan province (southeast Iraq), *Tylenchorhynchus clarus* and *T. zeae* were discovered around the rhizosphere of sugarcane and pumpkin, respectively. The morphological and morphometric data were provided for the recovered species. The morphological characters of both populations are in agreement with the type populations and other populations of them. To the best of our knowledge, this is the first report of these two species from Iraq, and a first report of the association of *T. zeae* with pumpkin. Molecular phylogenetic analyses of the Iraqi populations of *T. clarus* and *T. zeae* using the D2–D3 expansion segments of 28S rDNA and internal transcribed spacer (ITS) rDNA sequences using Bayesian inference (BI), showed they form maximally supported clades with other sequences of both species.

The cosmopolitan nematode genus *Tylenchorhynchus* Cobb, 1913, is one of specious genera. Its representatives are migratory ectoparasites of a wide range of plant species (Siddiqi, 2000). It is morphologically similar to the genus *Bitylenchus* Filipjev, 1934. According to Gómez-Barcina *et al*. (1992), genus *Bitylenchus* is differentiated from *Tylenchorhynchus* in having areolated outer bands of lateral fields, a large post-anal intestinal sac containing intestinal granules and fasciculi, relatively more thickened cuticle at the female tail tip, and gubernaculum lacking a crest. These characters are described for several species belonging to both *Tylenchorhyncus* and *Bitylenchus* (Handoo, 2000). Some nematologists considered *Bitylenchus* as a junior synonym of *Tylenchorhynchus*. Siddiqi (2000) listed 105 valid species under the genus *Tylenchorhynchus*. Geraert (2011) considered *Bitylenchus* as a junior synonym of *Tylenchorhynchus* and placed 133 species under the genus. Subsequently, *T. bambusi* Singh, Lal, Rathour & Ganguly, 2010, *T. mediterraneus* Handoo, Palomares-Rius, Cantalapiedra-Navarrete, Liébanas, Subbotin & Castillo, 2014, and *T. iranensis* Azimi, Mahdikhani-Moghadam, Rouhani & Rajabi Memari, 2016 were described from India, Spain, and Iran, respectively. In studies on integrative taxonomy of the genera *Bitylenchus* and *Tylenchorhynchus*, these two genera were separated from each other and considered as valid genera (Ghaderi *et al*., 2014; Handoo *et al*., 2014; Hosseinvand *et al*., 2020; Azizi *et al*., 2022).

Stephan *et al*. (1985) have reported 20 genera and 43 species of plant-parasitic nematodes from vineyard soils of Iraq. Morphological and morphometric data were however not provided for any of the reported species in previous study. *Tylenchorhynchus brassicae* Siddiqi, 1961 and *T. mashhoodi* Siddiqi and Basir, 1959 are two species of the genus *Tylenchorhynchus* that were included in this report.

During a survey on the plant-parasitic nematodes of the Misan province, southeast Iraq, *T. clarus* Allen, 1955 and *T. zeae* Sethi and Swarup, 1968 were recovered. According to published literature, this is the first report of these two species from Iraq. Thus, the present study aims to characterize the Iraqi populations of the two recovered species based on morphological and morphometric characteristics. Furthermore, molecular data of two D2–D3 expansion segments of 28S rDNA and ITS rDNA were used to study the phylogenetic relationships of the recovered species with other relevant species and genera.

## Materials and Methods

### Nematode extraction and morphological observations

Several soil samples were collected from the rhizosphere of sugarcane (*Saccharum officinarum* L.) and pumpkin (*Cucurbita moschate* L.) in Misan province, Iraq. The centrifugal flotation technique (Jenkins, 1964) and the tray method (Whitehead and Hemming, 1965) were used to extract the nematodes from soil samples. The collected specimens were killed in a hot 4% formaldehyde solution and transferred to anhydrous glycerin according to the method discussed in the study of De Grisse (1969). Observations and measurements were conducted using a Leitz SM-LUX light microscope (Leitz Corporation, wetzlar, Germany) equipped with a drawing tube. Some of the specimens were photographed using an Olympus Corporation, Tokyo, Japan DP12 digital camera attached to an Olympus BX51 light microscope (Olympus Corporation, Tokyo, Japan).

#### Taxonomic framework

The taxonomic framework proposed by Siddiqi (2000) was followed in this study, which considered *Bitylenchus*, *Sauertylenchus* Sher, 1974, and *Tylenchorhynchus* as valid genera.

#### DNA extraction, PCR and sequencing

For molecular analyses, single female specimens were picked out, examined in a drop of distilled water on a temporary slide under the light microscope, transferred to 5 μl of TE buffer (10 mM Tris-Cl, 0.5 mM EDTA; pH 9.0) on a clean slide, and then crushed using a cover slip. Each suspension was collected by adding 15 μl TE buffer. The DNA samples were stored at –20°C until they were used as PCR templates. Primers for LSU rDNA D2–D3 amplification were forward primer D2A (5'-ACAAGTACCGTGAGGGAAAGT-3') and reverse primer D3B (5'-TCGGAAGGAACCAGCTACTA-3') (Nunn, 1992). Primers for amplification of ITS rDNA were forward primer rDNA1 (5'-TTGATTACGTCCCTGCCCTTT-3') and reverse primer rDNA1.58S (5'-ACGAGCCGAGTGATCCACCG-3') (Subbotin *et al*., 2000). The 30 μl PCR mixture contained 10 μl of distilled water, 15 μl of Master Mix 2X (Ampliqon, Odenese, Denmark), 1 μl of each primer (10 pmol/μl), and 3 μl of DNA template. The thermal cycling program for amplification of both markers was as follows: denaturation at 95°C for 6 min, followed by 35 cycles of denaturation at 94°C for 30 s, annealing at 52.5°C (LSU D2–D3 primers) / 54.7°C (ITS rDNA primers) for 30 s, and extension at 72°C for 60 s. A final extension was performed at 72°C for 10 min. Amplification success was evaluated by electrophoresis on 1% agarose gel. The PCR products were purified using the QIAquick PCR purification kit (Qiagen, Hilden, Germany) following the manufacturer’s protocol and sequenced directly using the PCR primers with an ABI 3730XL sequencer (Bioneer Corporation, Daejeon, South Korea). The newly obtained sequences of the studied species were deposited into the GenBank database (accession numbers ON651683, ON651684, ON651685, ON651686 for LSU D2–D3, and ON667938, ON667939 for ITS rDNA).

**Table 1.** Morphometrics of *Tylenchorhynchus clarus* Allen, 1955 and *T. zeae* Sethi and Swarup, 1968 from Misan province, Iraq. All measurements are in micrometer and in the form: mean ± s.d. (range).

**Characters**	***Tylenchorhynchus clarus***	***Tylenchorhynchus zeae***	
	**Females**	**Females**	**Males**
n	14	13	5
L	459.1 ± 34.9 (414–523)	534.2 ± 15.5 (501–570)	483.5 ± 23.9 (455–521)
a	28.4 ± 1.2 (25.7–29.7)	28.9 ± 0.7 (25.7–32.2)	31.2 ± 0.4 (30.5–31.9)
b	4.6 ± 0.4 (4.0–5.4)	4.7 ± 0.1 (4.6–5.0)	4.7 ± 0.3 (4.3–5.2)
c	16.4 ± 1.5 (14.6–19.4)	16.7 ± 2.7 (14.0–20.4)	16.9 ± 2.1 (14.8–18.2)
c'	2.5 ± 0.2 (2.1–2.8)	2.2 ± 0.3 (1.9–2.6)	2.2 ± 0.2 (2.1–2.4)
V	57.5 ± 3.6 (53.5–59.8)	57.4 ± 3.7 (55.4–61.9)	–
Lip region height	3.1 ± 0.2 (2.8–3.2)	3.0 ± 0.2 (2.8–3.2)	2.7 ± 0.4 (2.4–3.3)
Lip region width	6.6 ± 0.4 (6.4–7.2)	5.7 ± 0.7 (5.5–7.3)	5.4 ± 0.4 (4.9–6.0)
DGO	1.9 ± 0.2 (1.3–2.4)	3.1 ± 0.1 (2.9–3.3)	3.1 ± 0.1 (2.9–3.3)
MB	51.2 ± 1.4 (46.6–55.8)	54.5 ± 1.9 (48.1–56.2)	53.3 ± 0.9 (52.2–54.7)
Stylet length	16.8 ± 1.6 (15.4–18.9)	18.2 ± 0.8 (16.7–19.2)	16.9 ± 0.6 (15.8–18.2)
Pharynx length	105.2 ± 4.8 (98–120)	113.3 ± 4 (106–117)	100.9 ± 6.4 (91–107)
Anterior end to excretory pore	76.9 ± 2.8 (72.8–86.8)	88.1 ± 3.2 (82–92)	76.2 ± 7.3 (70.5–87.1)
Maximum body width	16.2 ± 0.9 (15.2–17.6)	16.2 ± 0.4 (15.7–16.6)	15.4 ± 0.7 (14.9–16.6)
Anal body width	11.4 ± 0.9 (10.4–13.6)	12.4 ± 0.4 (11.6–14.2)	9.6 ± 0.9 (9.1–10.7)
Vulval body width	15.5 ± 0.9 (14.4–17.8)	17.5 ± 1.2 (14.6–19.2)	–
Tail length	29.1 ± 1.6 (27.5–33.2)	30.8 ± 2.9 (25.2–38.2)	26.8 ± 2.6 (24.2–33.7)
Tail annuli	15.5 ± 1.3 (14–16)	17.5 ± 1.1 (16–21)	–
Spicule length	–	–	17.5 ± 0.2 (16.1–18.9)
Gubernaculum	–	–	7.6 ± 0.3 (7.3–9.1)

#### Phylogenetic analyses

The newly obtained sequences of the D2–D3 fragments of LSU rDNA and ITS rDNA and additional sequences of relevant species were selected after the nucleotide basic local alignment search tool (BLASTn, National Center for Biotechnology Information, Bethesda, Maryland, United States) search. The sequences were aligned by Clustal X version 2 using the default parameters (Larkin *et al*., 2007). The outgroup taxa were chosen according to a previous study (Abdolkhani and Azimi, 2021). The editing of both alignments was performed manually in the MEGA7 program (Kumar *et al*., 2016). The base substitution model was selected using MrModeltest2 (Nylander, 2004) based on the Akaike information criteria. A general time reversible model, including among-site rate heterogeneity and estimates of invariant sites (GTR + G + I), was used in both phylogenies. The Bayesian analysis was performed to infer the phylogenetic trees using MrBayes v3.1.2 (Ronquist and Huelsenbeck, 2003), running the chains for four million generations. After discarding burn-in samples and evaluating convergence, the remaining samples were retained for further analyses. The Markov chain Monte Carlo (MCMC) method within a Bayesian framework was used to determine equilibrium distribution and help estimate the posterior probabilities of the phylogenetic trees (Larget and Simon, 1999) using the 50% majority rule. Bayesian posterior probability (BPP) values >0.50 are given on appropriate clades. The output files of the phylogenetic program were visualized using Dendroscope v3.2.8 (Huson and Scornavacca, 2012) and were digitally drawn in CorelDRAW software version 17 (Corel Corporation, Ottawa, Canada).

## Results

### Taxonomy

**Figure 1:**
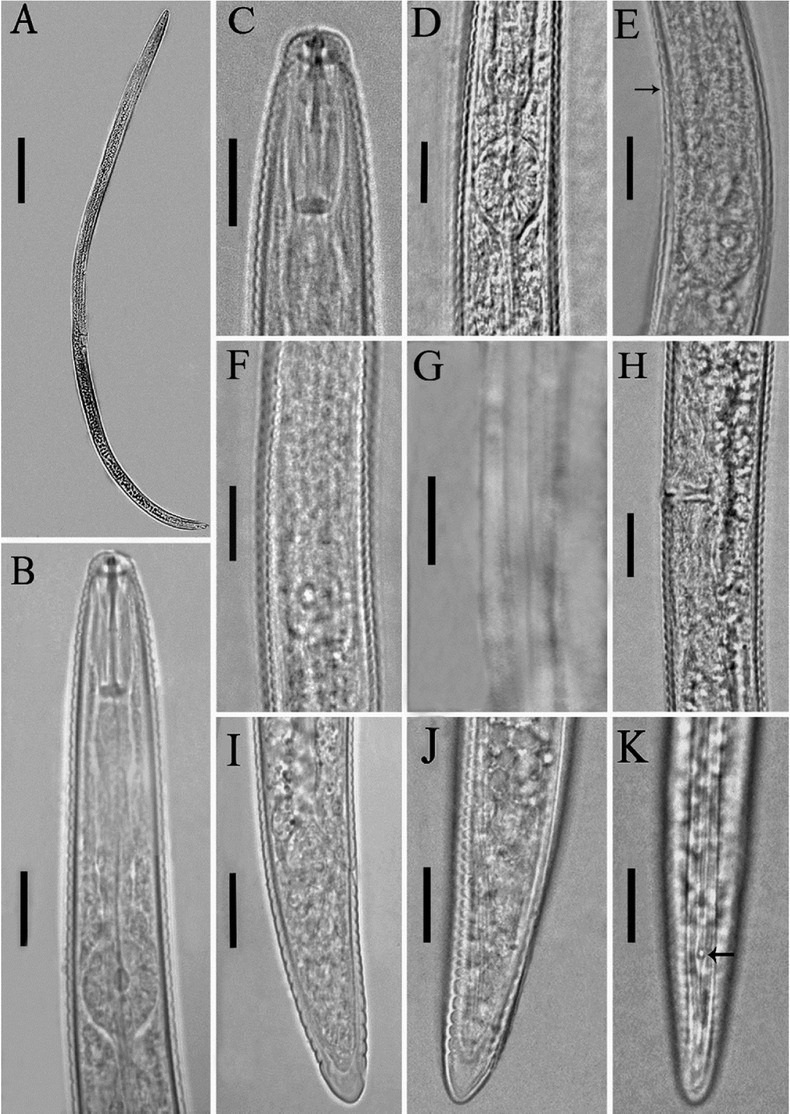
Light photomicrographs of *Tylenchorhynchus clarus* Allen, 1955 from Iraq. **(A)** Entire body; **(B, C)** Anterior body region; **(D)** Median bulb; **(E, F)** Terminal bulb (the arrow indicates the excretory pore); **(G)** Lateral field at mid-body; **(H)** Vulval region; **(I–K)** Posterior body region (the arrow indicates the phasmid). (Scale bars: A, 50 μm; B–K, 10 μm).

*Iraqi population of Tylenchorhynchus clarus* (Fig. 1 and Table 1)

## Description

### Female

Body arcuate ventrally to open C shaped after heat fixation. Cuticle annuli 1.6–2.3 μm wide at mid-body. Lateral field 6.0–7.8 μm wide, with four incisures along the body, outer lines slightly crenate. Lip region continuous to slightly offset from the body, bearing four to five annuli, cephalic framework weak. Stylet about 2.5 times lip region width, knobs rounded, their anterior surfaces flattened to slightly concave, 3–4 μm across. Median pharyngeal bulb elliptical, basal bulb elongate pyriform, overlapping intestine for 2–3 μm in most specimens. Hemizonid usually one to three annuli anterior to excretory pore. Cardia well developed. Intestinal fasciculi present in the intestinal region. Post-anal intestinal sac absent. Reproductive system didelphic–amphidelphic, both genital branches almost equally developed, ovaries straight, vagina about half corresponding body diameter long, vulva a transverse slit, spermatheca underdeveloped. Tail conoid, its terminus smooth, slightly narrowing, hyaline portion 3.5–5.0 μm thick. Phasmids 8–12 μm behind the anus.

#### Male

Not found.

### Remarks

The general morphology of the recovered population of the species closely resembles that of the type population (Allen, 1955) and other populations from Jordan (Hashim, 1983), southern Spain (Castillo *et al*., 1991), Spain and USA (Handoo *et al*., 2014), and Iran (Ghaderi and Karegar, 2016). Compared with the Egyptian population reported by Elmiligy (1969), the body length is shorter (414–523 *vs* 565–610 μm) and the number of tail annuli is less (14–16 *vs* 19–20 μm). Compared to the data given by Geraert (2011), no remarkable differences were observed.

The presently studied species was recovered from the rhizosphere of sugarcane in Misan province, southeast Iraq (GPS coordinates: 31°35'22.48"N, 47°12'31.98"E). The present study is the first to report *T. clarus* from Iraq.

#### Iraqi population of Tylenchorhynchus zeae

**Figure 2:**
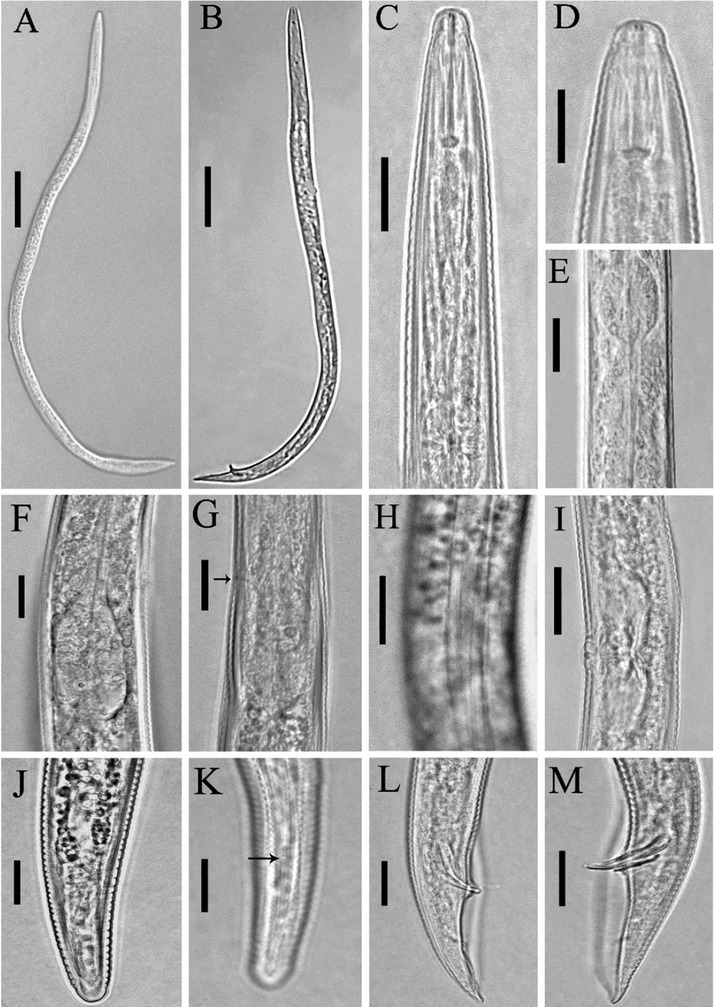
Light photomicrographs of *Tylenchorhynchus zeae* Sethi and Swarup, 1968 from Iraq. **(A, C–K)** Female. **(A)** Entire body; **(C, D)** Anterior body region; **(E)** Median bulb and isthmus; **(F, G)** Terminal bulb (the arrow indicates the excretory pore); **(H)** Lateral field at mid-body; **(I)** Vulval region; **(J, K)** Posterior body region (the arrow indicates the phasmid); **(B, L, M)** Male. **(B)** Entire body; **(L, M)** Posterior body region. (Scale bars: A and B, 50 μm; C–M, 10 μm).

(Fig. 2 and Table 1)

#### Description

##### Female

Body arcuate ventrally after heat fixation. Cuticle annuli 1.1–1.4 μm wide at mid-body. Lateral field with four incisures along the body, outer lines slightly crenate. Lip region slightly offset from the body, bearing four annuli, cephalic framework slightly sclerotized. Stylet about six times length of lip region, knobs rounded, their anterior surfaces flattened, 3.1–3.7 μm across. Median pharyngeal bulb elliptical, basal bulb pyriform to slightly cylindrical. Excretory pore about at level with the pharyngeal bulb. Cardia distinct. Post-anal intestinal sac absent. Reproductive system didelphic– amphidelphic, both genital branches almost equally developed, ovaries straight, spermatheca rounded, filled with rounded sperm, vagina about half body diameter long, vulva a transverse slit. Tail conoid to sub-cylindrical, its terminus widely rounded, smooth, hyaline portion 2.5–3.6 μm. Phasmids in the middle of the tail.

##### Male

General morphology is similar to that of female except for character states associated with sexual differences. Tail conoid, its tip pointed, enveloped with bursa. Spicules slightly curved ventrally. Gubernaculum well developed, about half the length of the spicules. Bursa 36.8–51.9 μm long.

#### Remarks

The general morphology of the recovered population of the species closely resembles that of the type population (Sethi and Swarup, 1968). However, the length of the tail is shorter (25.2–38.2 *vs* 46 μm). This species has already been reported from Spain (Handoo *et al*., 2014), Iran (Alvani *et al*., 2017), and China (Xu *et al*., 2020). There is no significant difference between these populations and the population recovered from Misan province (southeast Iraq) as part of the current study. Compared to the morphometric data ranges given by Geraert (2011), the Iraqi population has a slightly shorter tail (25.2–38.2 *vs* 46 mm).

The presently studied species was recovered from the rhizosphere of pumpkin in Misan province, southeast Iraq (GPS coordinates: 32°07'56.81"N, 46°43'05.75"E). The present study is the first to report the discovery of *T. zeae* in Iraq, and additionally represents the first report of its association with pumpkin.

#### Molecular characterization and phylogenetic relationships

##### D2–D3 fragments of 28S rDNA phylogeny

Three 718 nt long D2–D3 expansion segments of LSU rDNA (ON651683, ON651684, ON651685) were obtained for the Iraqi population of *T. clarus*. The BLAST search using these sequences revealed that they have 99.58% identity with another sequence of the same species (KJ461536). The sequence variation between the Iraqi population and this sequence amounted to five mismatches and one gap.

A 692 nt long partial sequence of the D2–D3 region with accession number ON651686 was obtained for the Iraqi population of *T. zeae*. The BLAST search using this sequence revealed that it has 99.86% identity with another sequence of the same species (KJ461565). The sequence variation between the Iraqi population and this sequence amounted to one mismatch.

**Figure 3:**
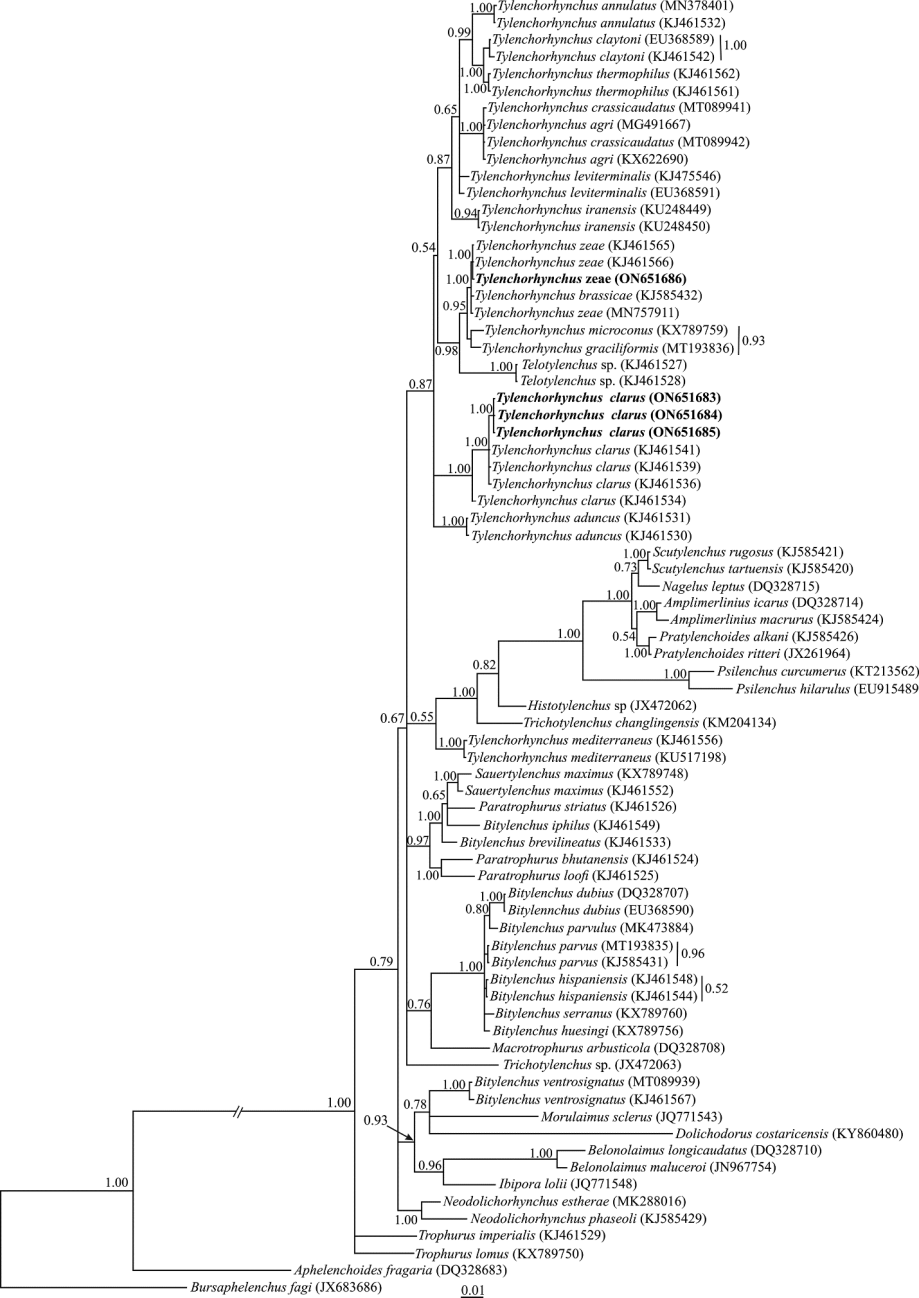
Bayesian 50% majority rule consensus tree inferred from analysis of the D2–D3 domains of the LSU rDNA sequences of Iraqi populations of *Tylenchorhynchus clarus* Allen, 1955 and *T. zeae* Sethi and Swarup, 1968 under the GTR + G + I model. Bayesian posterior probability values of >0.50 are given for appropriate clades. New sequences are indicated in bold.

A total of 74 sequences of Tylenchoidea Örley, 1880 and two sequences of Aphelenchoidea (Fuchs, 1937) Thorne, 1949 as outgroup taxa (DQ328683 and JX683686) were selected for the LSU phylogeny. This dataset comprised 797 total characters. The phylogenetic tree inferred using this dataset is presented in Figure 3. The newly generated sequences of the Iraqi populations of *Tylenchorhynchus clarus* and *T. zeae* have formed a maximally supported clade with other sequences of both species in this tree.

##### Partial ITS rDNA phylogeny

The sequencing of the ITS rDNA of the Iraqi population of *Tylenchorhynchus clarus* yielded one fragment with a 662 nt long sequence (ON667938). The BLAST search using this sequence revealed it has 99.80% identity (one mismatch and no gap) with another ITS sequence of the species (KJ461575).

A newly obtained 649 nt long partial sequence of the ITS rDNA with accession number ON667939 was generated for the Iraqi population of *T. zeae*. The BLAST search using this sequence revealed it has 99.69% identity with another ITS sequence of the species (MN757910). The sequence variation between the Iraqi population and this sequence amounted to two mismatches and no gap.

**Figure 4:**
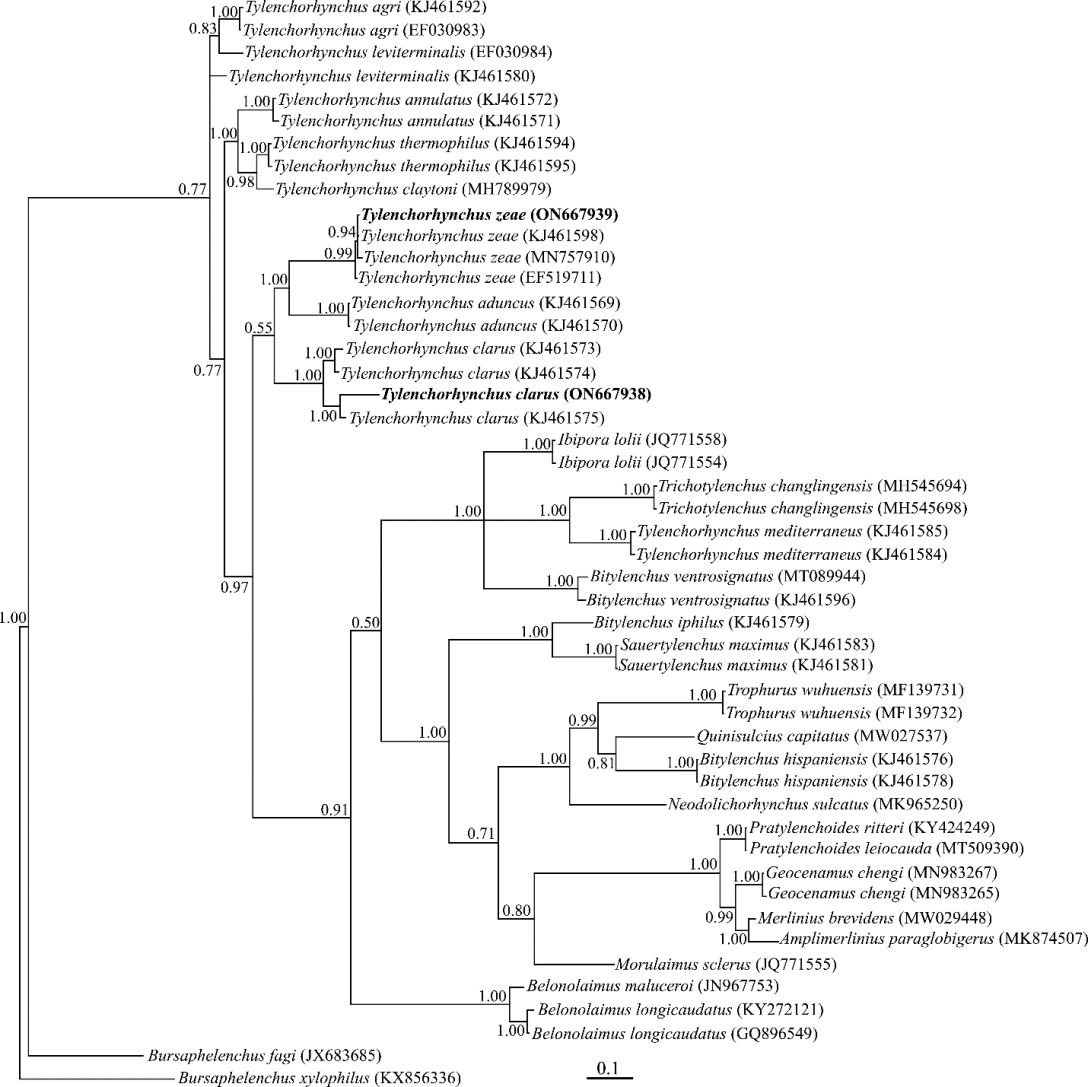
Bayesian 50% majority rule consensus tree inferred from analysis of the ITS rDNA of Iraqi populations of *Tylenchorhynchus clarus* Allen, 1950 and *T. zeae* Sethi and Swarup, 1968 under the GTR + G + I model. Bayesian posterior probability values of >0.50 are given for appropriate clades. New sequences are indicated in bold.

A total of 46 sequences of Tylenchoidea and two sequences of Aphelenchoidea as outgroup taxa (JX683685 and KX856336) were selected for ITS phylogeny. This dataset comprised 1,285 total characters. The phylogenetic tree inferred using this dataset is presented in Figure 4. The sequences of the Iraqi populations of *Tylenchorhynchus clarus* and *T. zeae* formed a maximally supported clade with other sequences of both species in this tree.

## Discussion

The objectives of this study were the morphological and molecular characterization of recovered populations of *Tylenchorhynchus clarus* and *T. zeae* from Iraq. In general, there are currently poor data on diversity of plant-parasitic nematodes in Iraq, and both reported species represent new records for the country.

Phylogenetic analyses and the subsequent alternative hypothesis tests based on the 28S rDNA by Handoo *et al*. (2014) showed the monophyly of the genus *Tylenchorhynchus sensu* Siddiqi (2000), although a representative of the genus *Telotylenchus* Siddiqi, 1960 was embedded inside the clade of *Tylenchorhynchus* species. Similar results have been achieved in some recent molecular phylogenies (Azimi *et al*., 2016; Abdolkhani and Azimi, 2021; Azizi *et al*., 2022). In the present 28S phylogeny, most of the included *Tylenchorhynchus* species + *Telotylenchus* sp. formed a clade and *Tylenchorhynchus mediterraneus* occupied a distant placement. This situation showed that the genus *Tylenchorhynchus* is polyphyletic.

The alternative hypothesis test by Handoo *et al*. (2014) using the ITS rDNA marker again corroborated the monophyly of the genus *Tylenchorhynchus*. In their original ITS tree, *T. mediterraneus* had occupied a distant placement. In the present study, the *Tylenchorhynchus* species were separated into different clades and *T. mediterraneus* formed a separate clade with other genera. This result represents that the genus is not monophyletic. Similar results were obtained in some recent phylogenies (Abdolkhani and Azimi, 2021; Sheikhzadeh *et al*., 2022).

A molecular phylogenetic study using the higher number of species of the closely related genera like *Bitylenchus*, *Paratrophurus* Arias, 1970, *Sauertylenchus*, and *Tylenchorhynchus* could result in a better phylogeny of these genera in future.

## Acknowledgments

The authors thank the Research Council of Shahid Chamran University of Ahvaz, Iran (Grant No. SCU. AP1400.638) for financial support.
